# Identifying temporal patterns in patient disease trajectories using dynamic time warping: A population-based study

**DOI:** 10.1038/s41598-018-22578-1

**Published:** 2018-03-09

**Authors:** Alexia Giannoula, Alba Gutierrez-Sacristán, Álex Bravo, Ferran Sanz, Laura I. Furlong

**Affiliations:** Research Program on Biomedical Informatics (GRIB), Hospital del Mar Medical Research Institute (IMIM), DCEXS, Universitat Pompeu Fabra, Barcelona, Spain

## Abstract

Time is a crucial parameter in the assessment of comorbidities in population-based studies, as it permits to identify more complex disease patterns apart from the pairwise disease associations. So far, it has been, either, completely ignored or only, taken into account by assessing the temporal directionality of identified comorbidity pairs. In this work, a novel time-analysis framework is presented for large-scale comorbidity studies. The disease-history vectors of patients of a regional Spanish health dataset are represented as time sequences of ordered disease diagnoses. Statistically significant pairwise disease associations are identified and their temporal directionality is assessed. Subsequently, an unsupervised clustering algorithm, based on Dynamic Time Warping, is applied on the common disease trajectories in order to group them according to the temporal patterns that they share. The proposed methodology for the temporal assessment of such trajectories could serve as the preliminary basis of a disease prediction system.

## Introduction

During the past years, there has been a growing interest in the study of disease associations in patients, known as comorbidities, due to their significant impact on health-care and clinical management. The term comorbidity can be defined as the co-occurrence of two or more conditions (e.g. diseases) in the same individual within a specified time period, with a long list of unfavourable outcomes, such as, decreased quality of life, higher cost of healthcare and higher mortality^1–3^. The progressive ageing of the population has led to an increasing number of patients with multiple coexisting (or subsequent) diseases during their clinical course, who are nowadays the rule rather than the exception^3,4^. A better understanding of comorbidities and their assessment within clinical, epidemiological and economic contexts is of major interest in order to improve disease management and reduce the associated healthcare costs.

The widespread use of electronic health records (EHR) and other clinical registries has expedited the massive collection of patient health information, thereby, enabling the implementation of population-based analyses of comorbidities^5–7^. Due to the nature of the available health data (e.g. short time span)^8–11^, the time factor has, typically, not been taken into account in most of the studies. However, by incorporating the time dimension into a comorbidity study and analysing the temporal onset of diseases in the patients, denoted hereafter as disease-history vectors, more complex disease patterns and their temporal characteristics can be revealed. The identification of time-related disease associations allows the prediction of the disease progression along time and can, potentially, facilitate the early diagnosis of other comorbid diseases. To the best of our knowledge, only a few large-scale disease trajectory studies have been reported so far^12,13^, in which, the time factor is accounted for by assessing the temporal directionality of comorbidity pairs and combining them, subsequently, into larger trajectories.

In this paper, the disease-history vectors of 643,358 patients are extracted from a regional Spanish health registry (corresponding to the province of Catalonia) and are temporally analysed, in order to investigate the most common comorbidities and their underlying time-dependent characteristics. This is achieved by representing the disease history of individual patients as time sequences of ordered disease diagnoses. Subsequently, pairwise comparisons are performed between these sequences for all patients of the dataset, according to a distance (similarity) metric that takes into account their time profiles. In this manner, ordered sequences of two or more diagnoses shared by at least two patients are extracted, which will be referred to, hereafter, as common disease trajectories. In the first part of the study, the significance of the common disease pairs (disease trajectories of length two) is assessed using pairwise statistical-significance tests. The temporal directionality of the corresponding associations is also assessed.

Afterwards, common trajectories of all lengths (number of diagnoses) are considered and shared time-dependent disease patterns are sought. For this reason, a novel unsupervised clustering algorithm is proposed, based on the *dynamic time warping* (DTW) technique, in order to group (cluster) the disease trajectories according to the temporal characteristics that they share. DTW is a powerful dynamic-programming technique for measuring similarities between two sequences that may vary in time or speed^14,15^. It has been successfully applied to speech analysis and other pattern recognition applications and herewith, we present its first implementation on patient disease trajectories. It will be shown that it can successfully group the disease trajectories under investigation, irrespective of the number of involved diseases and time scales. In this manner, meaningful clusters can be identified containing disease trajectories with similar time-dependent diagnosis patterns. Several clusters involving comorbidities already reported in the literature will be discussed from a perspective that, additionally, encompasses the time factor. Furthermore, other interesting disease associations and dynamics will be revealed. The results of this study are stratified according to sex and in this regard, several differences found between the male and female sub-populations will be pointed out.

Overall, the study of comorbidities within a time-dependent context is of crucial importance, as it is expected to shed light towards a better understanding of the progression of specific diseases and thus, to improve their clinical outcome.

## Results

### Pairwise comorbidity analysis

The total number of patients included in the CMBD database (see Methods section) was stratified according to gender. In the male sub-population (consisting of 303,722 patients), a total number of 12,905 comorbidity pairs (*d*_1_, *d*_2_) were identified using the Fisher’s exact test (see Methods), in which at least 10 patients shared the same two disease codes. After applying Bonferroni correction, 3,153 statistically significant comorbidity pairs were obtained using a significance level of 9.0 × 10^−8^. A list of the thirty most frequent statistically-significant comorbidities found in men and their corresponding *p*-values are shown in Table 1, where the temporal directionality is, also, indicated with an arrow. The most frequent disease association (encountered in 9,087 male patients irrespective of direction) was found to be between *chronic bronchitis* (ICD-9 code 491) and *other diseases of lung* (518), the latter encompassing various diseases, such as, *pulmonary collapse*, *interstitial emphysema*, *acute edema of lung*, etc.^16^. Other significant comorbidities include pairs of different respiratory diseases (e.g. *chronic bronchitis* (491) → *pneumonia* (486)), different types of cardiac diseases (e.g. *acute myocardial infarction* (410) → *ischemic heart disease* (414)), or combinations of a respiratory with a cardiac disease (e.g. *chronic bronchitis* (491) → *heart failure* (428)).

**Table 1:** Statistically significant comorbidity pairs in men. The thirty most frequent statistically significant pairwise comorbidities encountered in the male sub-population and their *p*-values. The arrow indicates preferred directionality (double arrow implies no preferred directionality). The total number of patients (#pat) sharing the diseases with either directionality is also shown. The comorbidity pairs are ordered according to the total number of patients.

Disease Association	#pat	*P*-value
Chronic bronchitis (491) → Other diseases of lung (518)	9,087	<4.9E-324
Cataract (366) → Chronic bronchitis (491)	7,749	1.9E-36
Chronic bronchitis (491) → Pneumonia (486)	7,091	<4.9E-324
Inguinal hernia (550) → Cataract (366)	6,692	3.0E-38
Acute myoc infarction (410) → Ischemic heart disease (414)	6,101	<4.9E-324
Chronic bronchitis (491) → Heart failure (428)	5,720	<4.9E-324
Cataract (366) → Other diseases of lung (518)	5,043	3.9E-9
Pneumonia (486) → Other diseases of lung (518)	4,546	<4.9E-324
Osteoarthrosis (715) → Cataract (366)	4,212	1.1E-66
Heart failure (428) → Other diseases of lung (518)	4,162	<4.9E-324
Other acute isch heart dis (411) → Ischemic heart disease (414)	4,026	<4.9E-324
Cataract (366) → Occl of cerebral arteries (434)	3,942	9.3E-18
Cataract (366) ↔ Ischemic heart disease (414)	3,915	1.6E-10
Cataract (366) → Acute myoc infarction (410)	3,902	1.2E-13
Hyperplasia of prostate (600) → Cataract (366)	3,820	4.0E-61
Cardiac dysrhythmias (427) → Heart failure (428)	3,792	<4.9E-324
Pneumonia (486) → Heart failure (428)	3,752	3.1E-258
Cataract (366) → Other dis urethra/urin tract (599)	3,578	7.6E-47
Acute myocardial infarction (410) → Heart failure (428)	3,421	<4.9E-324
Cataract (366) → Bladder cancer (188)	3,193	8.7E-13
Bladder cancer (188) → Other dis urethra/urin tract (599)	3,140	<4.9E-324
Ischemic heart disease (414) → Heart failure (428)	2,879	9.9E-190
Acute myocardial infarction (410) → Other acute isch heart dis (411)	2,870	<4.9E-324
Ac bronch (466) → Chronic bronchitis (491)	2,868	9.8E-161
Ac bronch (466) → Pneumonia (486)	2,854	2.7E-301
Cataract (366) → Ac bronch (466)	2,749	1.7E-120
Ac bronch (466) → Other diseases of lung (518)	2,728	2.6E-270
Diseases of pancreas (577) → Cholelithiasis (574)	2,660	<4.9E-324
Cataract (366) → Diabetes mellitus (250)	2,636	1.1E-22
Chronic bronchitis (491) → Emphysema (492)	2,619	<4.9E-324

A significant number of comorbidities (40%) within the thirty most populated disease associations reported in Table 1 for the male sub-population, involve *cataract* (366), such as, cataract with respiratory and cardiovascular diseases, as well as, with *osteoarthrosis* (715), *hyperplasia of the prostate* (600), *bladder cancer* (188) and *diabetes mellitus* (250), all disorders known to affect the eye, among others^16^. Diabetes is known to be an important risk factor for the formation of cataract and epidemiologic studies have demonstrated that cataracts are the most common cause of visual impairment in older-onset diabetic patients^17,18^. Strong associations of diabetes and smoking with cataract have been, also, found^19^. Both aforementioned cardiovascular risk factors are thought to induce cataract via oxidative damage of the proteins of the ocular lens^20^. Oxidative stress, which has been characterized as part of the ageing process, plays a significant role, also, in the development of heart disease. Therefore, cataractogenesis may be a marker for more generalized tissue damage and may be associated with increased risk of cardiovascular disease. Similarly, the pathogenic mechanism linking cataract with bladder cancer in men (see Table 1), could be hypothesized to be an insufficient antioxidative function, as commented in ref.^21^, where different types of cancer (including bladder cancer) were investigated in a nationwide population-based study and were found to occur with a higher incidence in patients with early-onset cataracts. Regarding the association of cataract with respiratory diseases, the underlying mechanisms are not fully understood, however it may be postulated that ageing is an important contributing factor for both types of diseases, similarly to the comorbidities previously discussed. Furthermore, there exist studies reporting an increased risk of developing cataract after prolonged and high doses of inhaled corticosteroids, often administered in patients with *Chronic Obstructive Pulmonary Disease* (COPD)^22^. As discussed in ref.^16^, eye disorders, including cataract, frequently constitute the first visible clinical manifestation of a variety of systemic disorders, such as, those presented previously.

In the female sub-population (339,636 women in total), 3,864 statistically-significant disease pairs (Bonferroni corrected *p*-value < 9.2 × 10^−8^) were identified and the thirty most frequent are shown in Table 2. As it can be observed, the *Osteoarthrosis* (715) → *Cataract* (366) pair is ranked first, shared by more than twofold patients compared to the corresponding association found in men. A more detailed analysis of osteoarthrosis and the associated trajectories in women will be presented in the following section. Other frequently observed comorbidities of the female sub-population are between different types of cardiovascular diseases, respiratory diseases, or a combination of these, similarly to the male sub-population. A comparison of the prevalence of several diseases or groups of diseases between men and women can be found in Supplementary Table 1. Cataract is a highly prevalent disease in both sub-populations (22% in men and 23% in women) and it also appears in a large part (50%) of the associations listed in Table 2, linked with cardiovascular, respiratory, musculoskeletal diseases, as well as, with diabetes and breast cancer. With respect to the latter, increased risk of cataract has been reported in women that received a specific anti-estrogen medication (tamoxifen) to treat breast cancer^23^.

**Table 2:** Statistically significant comorbidity pairs in women. The thirty most frequent statistically significant pairwise comorbidities encountered in the female sub-population and their *p*-values. The arrow indicates preferred directionality (double arrow implies no preferred directionality). The total number of patients (#pat) sharing the diseases with either directionality is also shown. The comorbidity pairs are ordered according to the total number of patients.

Disease Association	#pat	*P*-value
Osteoarthrosis (715) → Cataract (366)	10,665	<4.9E-324
Ac bronch (466) → Heart failure (428)	5,528	<4.9E-324
Heart failure (428) → Other diseases of lung (518)	5,067	<4.9E-324
Acquired deformities of toe (735) → Cataract (366)	4,943	9.1E-22
Cardiac dysrhythmias (427) → Heart failure (428)	4,860	<4.9E-324
Cataract (366) → Cholelithiasis (574)	4,810	4.5E-11
Cataract (366) → Ac bronch (466)	4,321	2.1E-34
Cataract (366) → Cardiac dysrhythmias (427)	4,141	1.4E-22
Cataract (366) → Occlusion of cerebral arteries (434)	3,798	6.2E-36
Ac bronch (466) → Other diseases of lung (518)	3,781	<4.9E-324
Cataract (366) → Other diseases of lung (518)	3,681	4.E-16
Cataract (366) → Other dis urethra and urinary tract (599)	3,342	8.9E-40
Pneumonia (486) ↔ Heart failure (428)	3,199	<4.9E-324
Diseases of pancreas (577) → Cholelithiasis (574)	3,156	<4.9E-324
Cataract (366) → Pneumonia (486)	3,058	1.1E-14
Mononeuritis upp. limb/multiplex (354) → Cataract (366)	3,002	3.8E-9
Ac bronch (466) → Pneumonia (486)	2,940	<4.9E-324
Acute myocardial infarction (410) → Heart failure (428)	2,721	<4.9E-324
Heart failure (428) → Hypertensive heart disease (402)	2,710	<4.9E-324
Heart failure (428) → Other dis urethra and urinary tract (599)	2,689	4.4E-167
Acquired deformities of toe (735) → Osteoarthrosis (715)	2,648	9.3E-157
Chronic bronchitis (491) ↔ Heart failure (428)	2,607	<4.9E-324
Chronic bronchitis (491) → Other diseases of lung (518)	2,601	<4.9E-324
Varicose veins lower extrem (454) → Cataract (366)	2,581	7.1E-131
Cataract (366) → Diabetes mellitus (250)	2,546	9.0E-24
Other hernia abdom (no obstr/gangr) (553) → Cataract (366)	2,474	5.0E-14
Cataract (366) ↔ Malignant neoplasm of female breast (174)	2,338	1.1E-53
Genital prolapse (618) → Cataract (366)	2,309	2.8E-53
Heart failure (428) → Occlusion of cerebral arteries (434)	2,273	2.6E-176
Pneumonia (486) → Other diseases of lung (518)	2,260	<4.9E-324

### Clustering of common disease trajectories using DTW

The statistically-significant disease pairs previously presented, together with the extracted common disease trajectories of lengths greater than two, were clustered using the proposed DTW technique in order to reveal temporal disease patterns. For the sake of simplicity, only common disease trajectories of lengths between 2 and 6 are considered, although trajectories of length up to 11 and 9 were obtained for men and women, respectively. A cut-off value for the minimum number of patients was applied to each trajectory length, in order to reduce the total number of trajectories to be clustered (see Supplementary Table 2). Pairs with statistically significant (preferred) directionality were included (*p*-values < 0.05 in the binomial test for directionality), while in the case of no preferred directionality (*p*-values ≥ 0.05), both directions were considered. A total number of 10,245 and 7,553 trajectories were, finally, plugged into the DTW clustering algorithm for male and female sub-populations, respectively. A threshold of 1,500 patients was empirically selected for the DTW clustering, as a trade-off between unnecessary merging and fragmentation of clusters (see Methods section).

### Retrieved clusters in men

In the male sub-population, 734 clusters were obtained, from which, only 199 were regarded as sufficiently large, i.e. containing ≥ 10 common disease trajectories (see Supplementary Table 3). For the sake of simplicity, only these 199 highly populated clusters were reviewed and, in some cases, those that shared similar patterns were combined into larger clusters for visualization purposes. A generic description of diseases (at the ICD-9 highest group level) within the twenty most populated clusters obtained for men is provided in Table 3. It can be observed that the first most populated and largest cluster (48,874 patients and 304 trajectories) comprises, almost in its totality (99.7%), trajectories with *diseases of the respiratory system* (codes 460–519), while the second most populated cluster (40,196 patients) is composed of *diseases of the circulatory system* (codes 390–459). Both classes of diseases are highly prevalent in the male sub-population of our dataset (37.9% and 31.8%, respectively), significantly surpassing the corresponding prevalence in the female sub-population, as reported in Supplementary Table 1. Other highly populated clusters were retrieved using the proposed methodology, involving different disease groups. In Supplementary Table 4, the aforementioned clusters are further decomposed into sub-groups of diseases, where a more detailed distribution of the diseases within each cluster is provided.

**Table 3:** The twenty most populated clusters extracted using DTW for the male sub-population. High-level (ICD-9 coding) description of the involved disease groups is provided for each cluster. The number of trajectories (#traj) and total number of patients (#pat) of each cluster is also listed. The clusters are ordered according to the total number of patients.

#traj	#pat	High-level disease group distribution
304	48,874	Dis Respir Sys (99.7%) Dis Circul Sys (0.3%)
162	40,196	Dis Circul Sys (100.0%)
132	22,437	Dis Genitour Sys (63.2%) Dis Digest Sys (36.8%)
238	18,648	Dis Respir Sys (51.5%) Dis Circul Sys (48.5%)
155	17,732	Dis Circul Sys (60.7%) Dis Respir Sys (39.3%)
221	16,961	Dis Nerv Sys & Sense Org (34.1%) Dis Circul Sys (65.9%)
192	13,557	Dis Circul Sys (58.4%) Dis Genitour Sys (27.1%) Dis Digest Sys (14.5%)
142	12,700	Dis Nerv Sys & Sense Org (31.7%) Dis Respir Sys (67.9%) Dis Circul Sys (0.4%)
45	11,379	Dis Nerv Sys & Sense Org (45.3%) Dis Digest Sys (38.7%) Dis Genitour Sys (16.0%)
61	10,191	Dis Digest Sys (46.0%) Dis Respir Sys (54.0%)
233	10,096	Dis Respir Sys (98.5%) Dis Digest Sys (1.4%) Dis Circul Sys (0.1%)
223	9,732	Dis Circul Sys (99.3%) Dis Respir Sys (0.7%)
51	9,405	Dis Nerv Sys & Sense Org (100.0%)
37	8,298	Dis Nerv Sys & Sense Org (41.7%) Dis Respir Sys (55.2%) Dis Digest Sys (3.1%)
63	8,243	Neoplasms (38.4%) Dis Genitour Sys (54.1%) Dis Digest Sys (7.6%)
57	7,871	Dis Digest Sys (90.3%) Dis Genitour Sys (8.9%) Dis Respir Sys (0.8%)
120	7,292	Dis Circul Sys (68.3%) Dis Nerv Sys & Sense Org (31.7%)
64	7,249	Dis Digest Sys (92.5%) Dis Genitour Sys (7.5%)
23	6,936	Dis Nerv Sys & Sense Org (43.4%) Neoplasms (56.6%)
56	6,748	Dis Genitour Sys (43.9%) Dis Respir Sys (55.4%) Dis Digest Sys (0.7%)

### Respiratory/circulatory clusters in men

**Figure 1:**
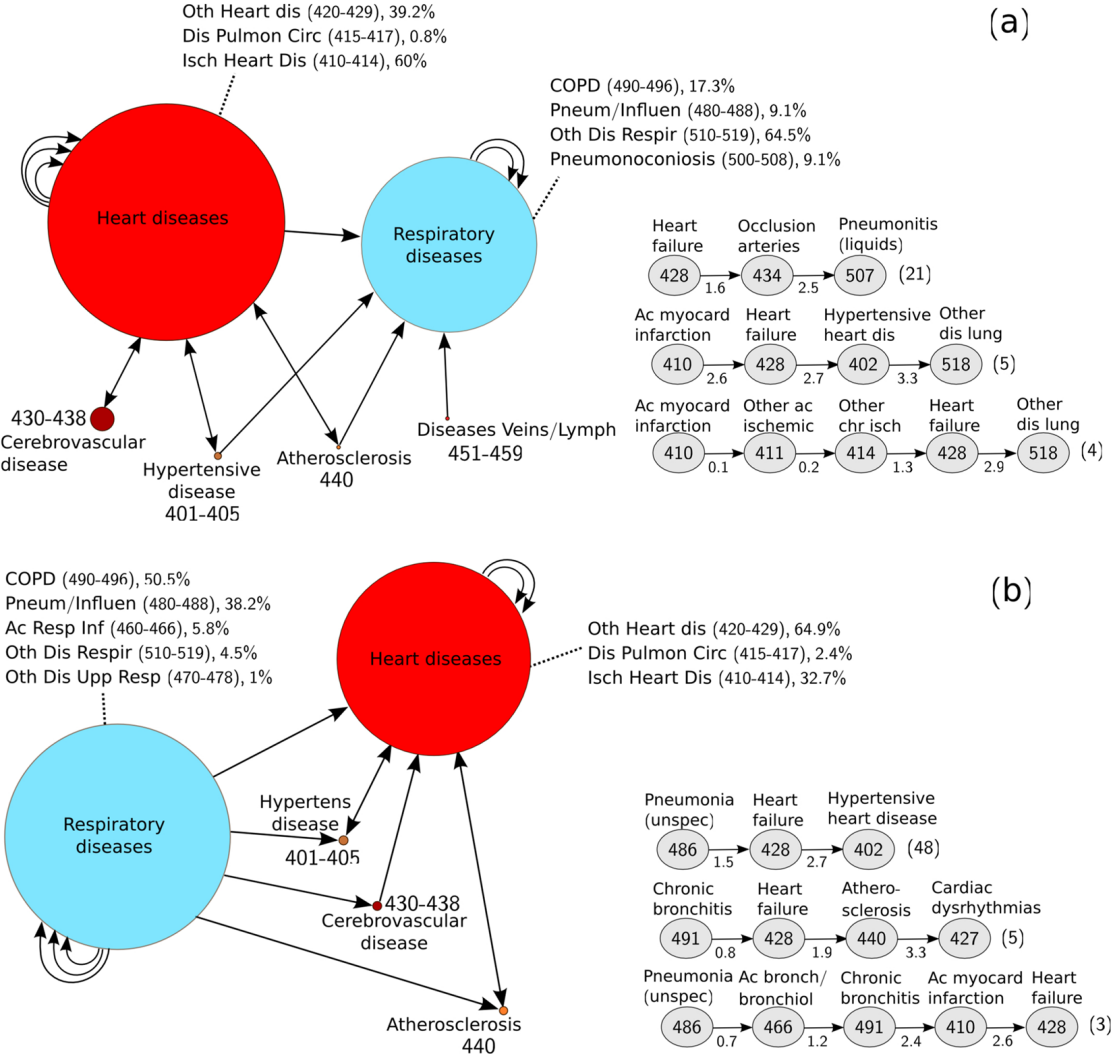
Schematic representation of two highly populated clusters (respiratory/circulatory). The (**a**) fifth and (**b**) fourth most populated clusters extracted for the male sub-population, associated with Table 3 (and Supplementary Table 4). The description of diseases is provided in each node. The nodes are drawn at a size relative to the frequency of appearance of the disease or group of diseases (a minimum node size corresponding to a frequency of 5% has been arbitrarily considered). Shorter and longer trajectories formed by connected nodes are contained in each cluster. Cyclic arrows indicate additional distinct diagnoses belonging to the same group of diseases (repetitions of the same disease are not permitted in a single trajectory). Examples of disease trajectories contained in each cluster are also provided on the bottom-right of the figure panels, together with the corresponding average times (indicated in years below each arrow) and average number of patients involved (shown at the end of each trajectory in parenthesis).

Two large and significantly populated clusters identified in men are visualized at a high level in Fig. 1a and b, both containing diseases of the circulatory and respiratory system that appear in different path combinations and frequencies. Figure 1a represents the fifth most populated cluster, involving 155 trajectories and 17,732 patients (also associated with the fifth entry of Table 3 and Supplementary Table 4). Most trajectories originate from one or more circulatory diseases (mainly cardiovascular diseases) and lead to one or more respiratory diseases. Cardiovascular diseases, including hypertension, are known to be a major comorbidity in patients suffering respiratory diseases, such as COPD^24,25^, increasing the mortality risk. At each time step, the corresponding diagnosis is different from all previous one, such that cyclic arrows indicate additional distinct diagnoses belonging to the same group/sub-group of diseases (note that repetitive disease diagnoses are not permitted in an individual trajectory, as explained in Methods). The sub-classification of the groups of diseases is shown in the corresponding nodes. The most frequent heart diseases (60%) observed in this cluster belong to the class of *ischemic heart disease* (codes 410–414), while the most frequent respiratory diseases (64.5%) belong to the group of *other diseases of the respiratory system* (codes 510–519). Cerebrovascular disease, hypertension or atherosclerosis can also occur before or after one or more diagnoses of a heart disease, followed by a respiratory one. Overall, the diseases of the circulatory system of this cluster represent ~60% of the total diseases found, while those of the respiratory system are ~40% (see fifth entry of Table 3).

Examples of individual trajectories found in this cluster can be also seen on the right-bottom panel of Fig. 1a, together with the average times (in years) between two consecutive diagnoses, averaged over the total number of patients (also indicated inside parenthesis). The illustrated trajectories do not, necessarily, follow the entire disease path of the cluster, in the sense that shorter or longer trajectories are permitted, given that similar temporal patterns are present. This is due to the fact that the DTW classification algorithm allows for the simultaneous clustering of trajectories of different durations and time scales, such that, e.g., a trajectory of three diseases spanning a period of 2.5 years (*Heart failure* (428) → *Occlusion of arteries* (434) → *Pneumonitis due to liquids* (507), see bottom-right panel of Fig. 1b) is clustered together with a longer trajectory that spans 2.9 years (*Acute myocardial infarction* (410) → *Other acute ischemic heart disease* (411) → *Other chronic ischemic heart disease* (414) → *Heart failure* (428) → *Other diseases of lung* (518)), since they follow the same key pattern (that is, a circulatory disease followed by a respiratory one, as described above).

A similar high-level representation of the fourth most populated cluster, which contains 238 trajectories and 18,648 patients, is shown in Fig. 1b and is composed of trajectories with diseases of the same groups as previously, but which, however, follow a reverse temporal key pattern, that is, a respiratory disease precedes a circulatory disease. The group and sub-group distributions of the respective diagnoses are, in this case, different (see fourth entry in Table 3 and Supplementary Table 4). The most prevalent heart diagnosis in this cluster is *heart failure* (428), which belongs to the class of *other forms of heart disease* (codes 420–429), while the most typical respiratory disease encountered is *chronic bronchitis* (491) of the COPD group (codes 490–496). Similarly, a diagnosis of hypertension, atherosclerosis or cerebrovascular disease can intervene with significantly lower frequency than the former diseases.

### Retrieved clusters in women

With respect to the female sub-population, the 164 largest clusters obtained out of a total of 703 (see Supplementary Table 3) were analysed. Description of the distribution of the groups of diseases contained in each cluster is provided in Table 4 and Supplementary Table 5. The largest extracted cluster, involving 374 trajectories and 58,672 patients, mainly comprises *complications of pregnancy*, *childbirth and the puerperium* (ICD-9 codes 630–379). Additional highly populated clusters involve *diseases of the respiratory* (460–519) and *circulatory* (390–459) *systems*, as well as, different combinations of them, similarly to the male sub-population, which are not shown due to lack of space. Although the majority of sub-groups of diseases of the circulatory system occur at a lower rate in women than in men, as shown in Supplementary Table 1, *hypertensive disease* (401–405) is slightly more prevalent in women. Respiratory diseases show a lower prevalence in the women sub-population, with COPD occurring in 5.1% of the cases (versus 11.2% in men). However, *asthma* (493) appears to be diagnosed with a higher frequency in women than in men of the examined dataset (2.7% versus 0.9%, respectively).

**Table 4:** The twenty most populated clusters extracted using DTW for the female sub-population. High-level (ICD-9 coding) description of the involved disease groups is provided for each cluster. The number of trajectories (#traj) and total number of patients (#pat) of each cluster is also listed. The clusters are ordered according to the total number of patients.

#traj	#pat	High-level disease group distribution
374	58,672	Compl Pregn Birth Puerp (98.6%) Dis Genitour Sys (1.4%)
220	29,781	Dis Respir Sys (100.0%)
301	28,588	Dis Circul Sys (99.9%) Dis Nerv Sys & Sense Org (0.1%)
160	20,430	Dis Nerv Sys & Sense Org (36.3%) Dis Circul Sys (62.9%) Dis Respir Sys (0.8%)
93	17,273	Dis Circul Sys (56.8%) Dis Respir Sys (42.0%) Dis Digest Sys (1.2%)
97	16,473	Dis Circul Sys (97.0%) Dis Respir Sys (3.0%)
51	14,427	Dis Musculosk Sys & Conn Tiss (51.4%) Dis Nerv Sys & Sense Org (47.8%) Dis Skin & Subcut Tis (0.7%)
75	13,234	Dis Musculosk Sys & Conn Tiss (100.0%)
68	12,598	Dis Digest Sys (95.9%) Dis Genitour Sys (4.1%)
130	12,373	Dis Respir Sys (43.4%) Dis Circul Sys (56.6%)
74	12,056	Dis Circul Sys (55.7%) Dis Genitour Sys (17.5%) Dis Digest Sys (26.8%)
42	11,133	Dis Nerv Sys & Sense Org (98.9%) Dis Circul Sys (1.1%)
62	7,843	Dis Musculosk Sys & Conn Tiss (41.1%) Dis Circul Sys (55.6%) Dis Respir Sys (2.6%) Dis Skin & Subcut Tis (0.7%)
41	7,652	Dis Nerv Sys & Sense Org (40.7%) Dis Digest Sys (49.1%) Dis Genitour Sys (10.2%)
45	7,010	Dis Genitour Sys (82.4%) Dis Digest Sys (17.6%)
135	6,464	Dis Circul Sys (58.0%) Dis Respir Sys (42.0%)
24	5,796	Dis Respir Sys (21.6%) Dis Genitour Sys (9.8%) Dis Circul Sys (25.5%) Dis Digest Sys (43.1%)
49	5,754	Dis Nerv Sys & Sense Org (44.4%) Dis Musculosk Sys & Conn Tiss (54.9%) Dis Skin & Subcut Tis (0.8%)
22	5,359	Dis Nerv Sys & Sense Org (47.1%) Dis Genitour Sys (51.0%) Compl Pregn Birth Puerp (2.0%)
63	5,332	Dis Nerv Sys & Sense Org (32.4%) Dis Respir Sys (67.6%)

### Specific-disease cluster paradigm in men: bladder cancer

**Figure 2:**
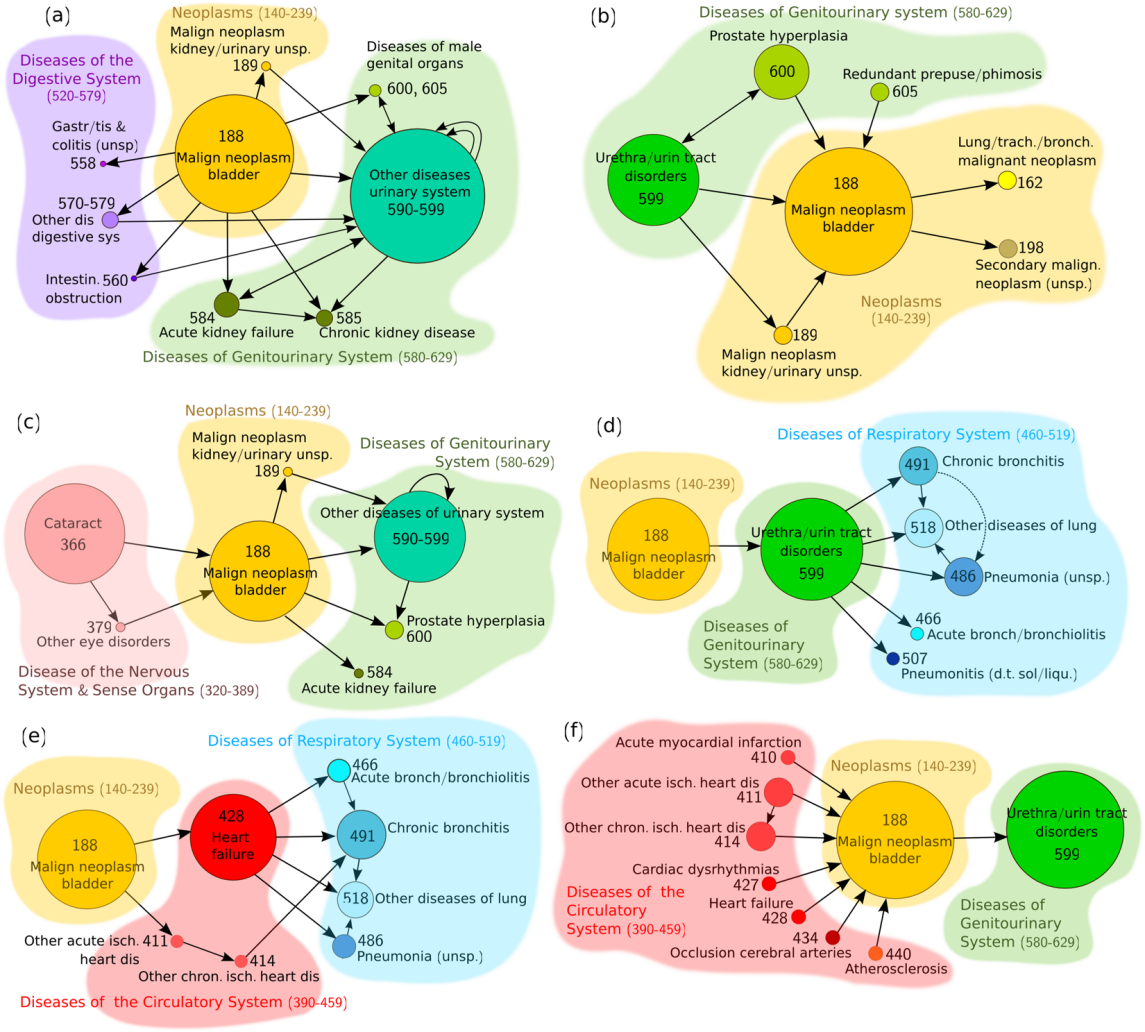
Clusters associated with bladder cancer. Six clusters containing disease trajectories associated with *bladder cancer* (ICD-9 code 188), extracted by the DTW clustering algorithm on the male sub-population. In each cluster, bladder cancer represented more than 20% of the total diagnoses. The nodes are drawn at a size relative to the frequency of appearance of the disease or group of diseases (a minimum node size corresponding to a frequency of 5% has been arbitrarily considered). Shorter and longer trajectories formed by connected nodes are contained in each cluster. Cyclic arrows indicate additional distinct diagnoses belonging to the same group of diseases (repetitions of the same disease are not permitted in a single trajectory).

*Bladder cancer* (188) accounts for about 5% of all new cancers in the US, being the fourth most common cancer in men^26^. In the present study, it was also found to be much more prevalent in the male population (with 5.2% as opposed to 0.7% in women) and it was ranked as the most frequent diagnosis of malignant neoplasm. Application of the proposed clustering method revealed 33 clusters (with ≥ 8 trajectories) in men, in which, the diagnoses of bladder cancer represented more than 5% of the total diagnoses. In Fig. 2, six of these clusters (in which bladder cancer constitutes ≥ 20% of the total diagnoses) are shown. The most populated cluster is illustrated in Fig. 2a and its trajectories involve, mainly, *diseases of the genitourinary system* (580–629), such as, *other diseases of the urinary system* (590–599), following bladder cancer. The aforementioned group of diseases is, primarily, composed of *other disorders of urethra and urinary tract* (599), as well as, *infections of kidney* (590), *other disorders of kidney and ureter* (593), *hydronephrosis* (591), *cystitis* (595), etc. Urinary tract infections have been, frequently, linked to bladder cancer, although there is controversy on whether they constitute a risk factor for its development or if they are a consequence of early bladder cancer before its diagnosis, rather than a cause of the disease^27,28^. In Fig. 2, *diseases of the urinary system* (590–599) or, in particular, *other disorders of urethra and urinary tract* (599) appear to be associated with bladder cancer in five out of the six of the clusters considered (Fig. 2a,b,c,d,f), with either directionality. In all cases, they represent a significant portion of the cluster diagnoses.

*Acute kidney failure* (584) and *chronic kidney disease* (585) are also found to come after a diagnosis of bladder cancer (Fig. 2a,c), the former known to be a common and severe complication in cancer-ill patients^29^. Furthermore, *hyperplasia of the prostate* (600) is quite frequently observed before or after bladder cancer (Fig. 2a,b,c). There has been evidence that patients suffering prostate enlargement are at increased risk for bladder cancer^30^, although in our study, no preferred directionality was found between these two diseases. In Fig. 2a, several *diseases of the digestive system* (520–579) form, also, part of the extracted trajectories, although at a lower rate than the *diseases of the genitourinary system*, with *cholelithiasis* (*gallstones*) (574) being the most frequent diagnosis. In the rest of the clusters, alternative disease patterns related to bladder cancer can be observed, such as, *diseases of the genitourinary system* leading to bladder cancer and other malignancies (Fig. 2b), or *cataract* (366) preceding bladder cancer (Fig. 2c), as well as, different path combinations associating bladder cancer with *diseases of the respiratory system* (460–519) (Fig. 2d), *diseases of the circulatory system* (390–459) (Fig. 2f) and combinations of circulatory and respiratory diseases (Fig. 2e). A number of potential determinants can be suggested to explain the above associations, such as, ageing, genetic susceptibility, common risk factors (e.g. smoking and physical inactivity), drug adverse effects, as well as, common biological pathways like oxidative stress and systemic inflammation^20,30–34^.

### Specific-disease cluster paradigm in women: osteoarthrosis

Osteoarthrosis (also known as osteoarthritis) is the most common form of arthritis and the major cause of physical disability in the elderly^35–37^. In the present study, osteoarthrosis exhibites a prevalence of 8.4% in the female sub-population, which is approximately 75% higher than that in the male one (see Supplementary Table 1). In fact, the entire group of *diseases of the musculoskeletal system and connective tissue* (710–739) is almost two-fold prevalent in women, in consistency with previous studies, where the majority of musculoskeletal conditions (such as, osteoarthrosis, rheumatoid arthritis and osteoporosis) were reported to affect women significantly more than men^36^. The prevalence of osteoarthrosis is increasing with age and thus, it is known to coexist with other chronic diseases^35,37,38^.

**Figure 3:**
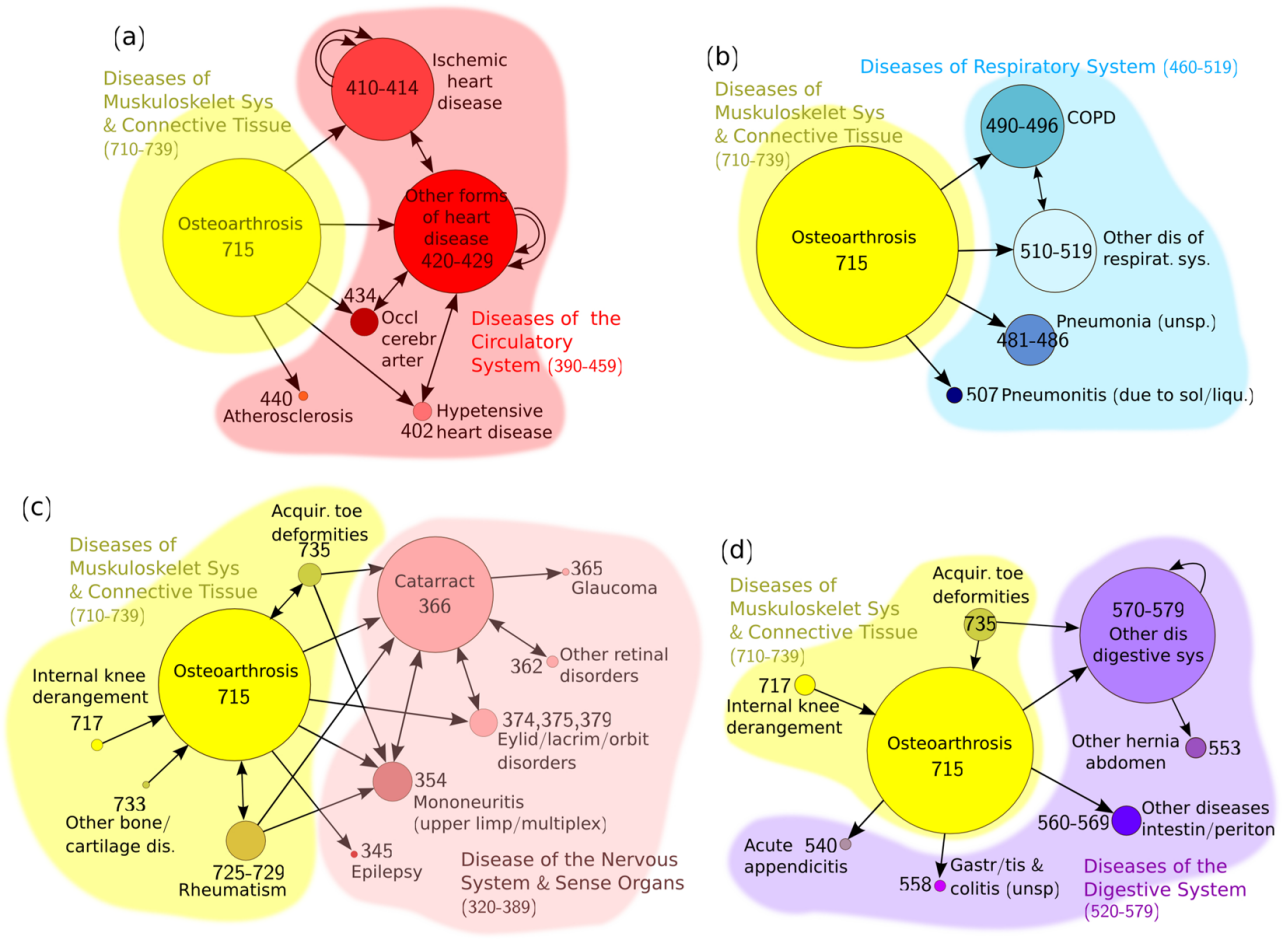
Clusters associated with osteoarthrosis. Four clusters containing disease trajectories associated with *osteoarthrosis* (ICD-9 code 715), extracted by the DTW clustering algorithm on the female sub-population. In each cluster, bladder cancer represented more than 20% of the total diagnoses. The nodes are drawn at a size relative to the frequency of appearance of the disease or group of diseases (a minimum node size corresponding to a frequency of 5% has been arbitrarily considered). Shorter and longer trajectories formed by connected nodes are contained in each cluster. Cyclic arrows indicate additional distinct diagnoses belonging to the same group of diseases (repetitions of the same disease are not permitted in a single trajectory).

A total of 16 clusters (with ≥ 8 trajectories) were extracted from the female sub-population, involving *osteoarthrosis* (715) with a rate ≥ 20% within each cluster. Four of these clusters are shown in Fig. 3. The cluster illustrated in Fig. 3a reveals associations of osteoarthrosis with *ischemic* and *other forms of heart disease* (group codes 410–414 and 420–429), as well as *hypertensive heart disease* (402), *atherosclerosis* (440) and *occlusion of cerebral arteries* (434). Traditional risk factors for the above diseases of the circulatory system include age, obesity, smoking and physical inactivity, which are also associated with the development and progression of osteoarthrosis, potentially highlighting shared pathophysiological processes^39–41^. A cluster containing trajectories that link osteoarthrosis with various diseases of the respiratory system, such as COPD, is demonstrated in Fig. 3b. Although little is known about this type of association, there are studies that report a large proportion of patients with osteoarthrosis that also suffer from a respiratory disease^35^. Trajectories linking osteoarthrosis and other *diseases of the musculoskeletal system and connective tissue* (710–739) with *diseases of the nervous system and sense organs* (320–389) are included in the cluster of Fig. 3c. The majority of these trajectories involve a prior diagnosis of osteoarthrosis (possibly associated, in addition, with *rheumatism* (725–729), or *internal knee derangement* (717), among other diseases), followed by *cataract* (366) and/or other disorders of the eye. In fact, *Osteoarthrosis* (715) → *Cataract* (366) was the most frequent statistically significant comorbidity found in women (Table 2). As discussed earlier, both diseases share common risk factors (e.g. age, smoking, etc.), but further studies should be performed in order to investigate possible common biological pathways. Finally, a cluster relating osteoarthrosis with *diseases of the digestive system* (520–579), mainly, *cholelithiasis* (574), *diseases of pancreas* (577) and *gastrointestinal hemorrhage* (578), is shown in Fig. 3d. The related gastrointestinal problems in patients with osteoarthrosis could be explained by the extensive use of non-steroidal anti-inflammatory drugs for the treatment of the symptoms of this disease^42^. Additional disease patterns encountered in the above clusters should be further studied.

## Discussion and Conclusions

A novel methodology was presented in this paper for the identification and temporal analysis of disease trajectories extracted from large cohort health datasets. To the best of our knowledge, only a few large-scale temporal comorbidity studies have been described so far in the scientific literature^12,13^, in which the time factor was only taken into account by assessing the temporal directionality between identified comorbidity pairs and longer trajectories were formed by combining those pairs based on overlapping diagnoses. In the present work, the entire temporal profile of the disease-history vectors was considered for all patients of the dataset by representing them as time sequences of ordered disease diagnoses and their pairwise similarities were assessed according to the global cost (or accumulated distance) metric (see the Methods section). In this manner, all possible common disease trajectories were identified and subsequently, analysed and clustered.

As a case study of the new methodology proposed, the disease history vectors of 303,722 men and 339,636 women were extracted from a health registry of a region of Spain and were temporally analysed as described above, in order to i) identify significant comorbidity pairs and determine their temporal directionality and ii) identify clusters of common disease trajectories of any length. The latter was achieved by applying a novel unsupervised clustering algorithm based on the dynamic time warping (DTW) technique, which was shown to group disease trajectories with no apparent temporal alignment according to the time-dependent disease patterns that they shared and irrespective of their time scale and duration. By stratifying the analysis according to gender, important differences in the two sub-populations were observed.

Many of the findings of the present study have been supported by previously published results, while additional disease associations and their time dynamics have been revealed. However, only a small part of the extracted clusters and disease associations have been presented and discussed, due to length limitations. Evidently, apart from the clusters presented in the results section, other smaller and less populated -yet important- clusters were also obtained containing interesting disease patterns that involved, for example, various types of neoplasms, hernia of abdominal cavity, non-infectious enteritis and colitis, anaemia, etc. Nevertheless, an extensive description of all the extracted clusters and identified disease patterns was out of the scope of this paper, whose aim was, primarily, to present a generic methodology that could be applied to any population-based epidemiological dataset in order to reveal complex time-dependent disease patterns, in addition to statistically significant pairwise comorbidities.

The results presented in this paper were, exclusively, based on hospitalized patient data and consequently, they should be interpreted accordingly. Possible errors in the diagnostic codes and admission dates, as well as incomplete data and inaccuracies, all inherent to the health dataset used, might cause certain variations in the resulting disease associations, as also discussed by several other authors^43,44^. Furthermore, some unexpected –according to the literature- temporal comorbidities identified (e.g., *Cataract* (366) → *Diabetes Mellitus* (250) or *Cataract* (366) → *Chronic Bronchitis* (491) in Table 1), could be possibly explained by the fact that some diseases share important risk factors (such as, ageing, smoking and/or obesity), thereby making difficult to define with accuracy which disease precedes or follows another. Furthermore, there may be cases of pre-existing diseases in patients with no clinical manifestations such that their diagnosis was made later in time. Confusion regarding the time directionality may, also, occur due to a medication therapy that is being followed for a particular disease, but which could, later, give rise to another disease as a side-effect. The elimination of secondary diagnoses adopted in this work, which is a common practice in this type of studies^12^, might also influence the extracted associations, such that a disease diagnosed as primary at a particular time instant, could be associated with another disease diagnosed as secondary at a previous point in time.

Furthermore, a simple Euclidean-like metric was used as a distance metric between two ICD-9 disease codes (equation (1)), although these do not constitute merely numerical variables. However, being ordinal ones and represented by numerical digits (3 digits in our study) that are hierarchically organized according to the ICD-9 disease classification system (www.icd9data.com) from 000 to 999 (forming numerical ranges of disease groups), they permit the use of such a distance metric to detect similarities of diseases at least at the group/subgroup level. Given the methodological focus of our work, together with the high amount of our input data (more than 10,000 and 7,000 trajectories to be clustered for men and women), this level of disease specificity was acceptable through the use of a simple, yet efficient, distance metric, such as the Euclidean distance. Moreover, the specificity of the identified disease associations within the extracted clusters can be further controlled using the value of the *threshold* of the iterative clustering algorithm (see Methods), which if appropriately selected, permits a fragmented but well homogeneous and compact clustering of trajectories, depending on the requirements, each time, of the case study under consideration. In fact, from the total of more than 700 clusters extracted for each gender, there could be found disease patterns at the highest-classification group level (e.g. associations between *respiratory* (460–519) and *circulatory* (390–459) diseases), as well as, at the sub-group level (e.g. *ischemic heart disease* (410–414) associated with *hypertensive disease* (401–405)) and also disease-specific associations (e.g. clusters involving comorbidities between *bladder cancer* (188) with other specific diseases or sub-groups of diseases, as seen in Fig. 3). Possible misclassifications of trajectories due to low Euclidean distances between diseases that, in fact, belonged to different disease groups could be corrected, at a great extent, by a post-refinement step, as explained in Methods.

Evidently, more sophisticated disease-similarity metrics can be, alternatively, adopted and directly incorporated into the clustering algorithm, such that, the generated clusters can reflect, for example, semantic, phenotypic, molecular, etc., similarities between diseases, depending on the distance metric employed each time. Finally, other factors could affect the resulting disease associations, such as the patients’ age, dietary habits, smoking, socioeconomic status, interacting medication, etc. For all the above issues, the interpretation of the obtained results should be carefully conducted. As mentioned earlier, the main objective of the paper was to present a novel methodology on the identification of complex time-dependent disease associations from a large patient registry, rather than an exhaustive clinical analysis.

Summarising, time is a crucial parameter in the assessment of comorbidities, as it permits the identification and study of disease associations and their directionalities. The presented methodology constitutes a novel time-analysis framework for cohort observational comorbidity studies, which can provide possible preliminary indications that could, potentially, assist in the study of the underlying disease mechanisms. By using a data mining approach, it was demonstrated how epidemiological patient health information, collected in routine clinical practice, could be exploited in order to discover important disease patterns and facilitate the prediction of the course of a disease given previous diagnoses, thereby, setting up the basis for the design of a preliminary disease prediction system. The proposed method can be applied to any type of health dataset and disease codification system. Including secondary diagnoses into the methodology, apart from the primary ones, constitutes another important challenge to be taken into account for a future work. In this case, the shared disease trajectories could be, likewise, retrieved and clustered in groups and the calculated time interval between two consecutive diagnoses (admitting, this time, values equal or larger than zero) would be an important factor for their interpretation, as well as, for a potential probability prediction algorithm in the context of time. Finally, further studies should be performed in order to test and verify the derived disease associations and temporal-directionality observations, through, for example. carefully designed biological experiments, clinical trials, etc., which altogether could help in explaining the aetiology of certain disease associations. In this way, a better understanding of diseases could be achieved, thereby leading, to more efficient and cost-effective clinical management and healthcare.

## Methods

### Data

For the objectives of this work, a Catalan-wide clinical registry was used (Conjunt Mínim Bàsic de Dades de Catalunya, CMBD), provided by the Catalan Institute of Health. CMBD contains de-identified demographic information such as age and gender data, as well as, disease diagnosis information from hospitalized patients, coded using the ICD-9 international coding system^45^. The dataset used in this study spans a time period of seven years (between 2004 and 2011) and includes 2,762,081 patients in total. In this study, all secondary diagnoses (SD) are excluded and only primary diagnoses (PD) are used. Furthermore, all “E” and “V” codes, associated with external causes of injury and supplementary classification, respectively, were also excluded and only patients with three or more (≥3) hospital visits are considered, thereby leading to a total number of 643,358 patients. The min, median and max number of hospital visits were calculated to be 3, 4 and 188 for men and 3, 4 and 78, for women. The proposed methodology was applied separately on men (303,722) and women (339,636). In Supplementary Fig. 1, the average age of the male and female sub-populations is illustrated for each respective year of diagnosis. Finally, ICD-9 disease codes at the 3-digit level were used^45^.

### Ethics

The data used in this study was provided by the Catalan Institute of Health in a completely anonymised format. The present study was approved by the PSMAR Research Ethics Committee (*PSMAR CEIC n° 2013/5270/I*) and all methods were performed in accordance with relevant guidelines and regulations.

### Extraction of common disease trajectories of various lengths

For each patient, a time sequence of ordered disease diagnoses (codes), known as disease-history vector, is extracted. A patient is considered to follow a disease-history vector only if he/she has been assigned the diagnoses strictly in the order specified by the vector. Only the first -in time- occurrence of each diagnosis is taken into account. Thus, a disease history vector *s* = {*d*_1_, *d*_2_, …, *d*_*N*_} for a specific patient, describes a sequence of diagnostic codes *d*_*k*_ recorded at discrete time instants *t*_*k*_ (*k* = *1, 2*, …, *N*), such that *s*(*t*_*k*_) = *d*_*k*_. Subsequently, lists of common disease trajectories of various lengths (i.e. number of distinct diagnoses) are obtained, by identifying those history vectors in which all diagnosis codes are shared with the exact same order by two or more patients. This is achieved by performing all possible pairwise comparisons between the history vectors of the dataset under consideration. Specifically, the disease history *s*_*i*_ = {*d*_1_, *d*_2_, …, *d*_*N*_} corresponding to patient *i* is compared with the disease history *s*_*j*_ = {*d*_1_, *d*_2_, …, *d*_*M*_} of patient *j*, according to a *local distance matrix D* of dimensions *N* × *M*, which is obtained by computing the squared difference between each pair of elements (codes) of the two vectors, i.e.,

1 $${D}_{ij(n,m)}={|{s}_{i}({t}_{n})-{s}_{j}({t}_{m})|}^{2},{\rm{for}}\,n\,=\,1,2,\,\ldots ,\,N,\,m=1,2,\,\ldots ,\,M$$

**Figure 4:**
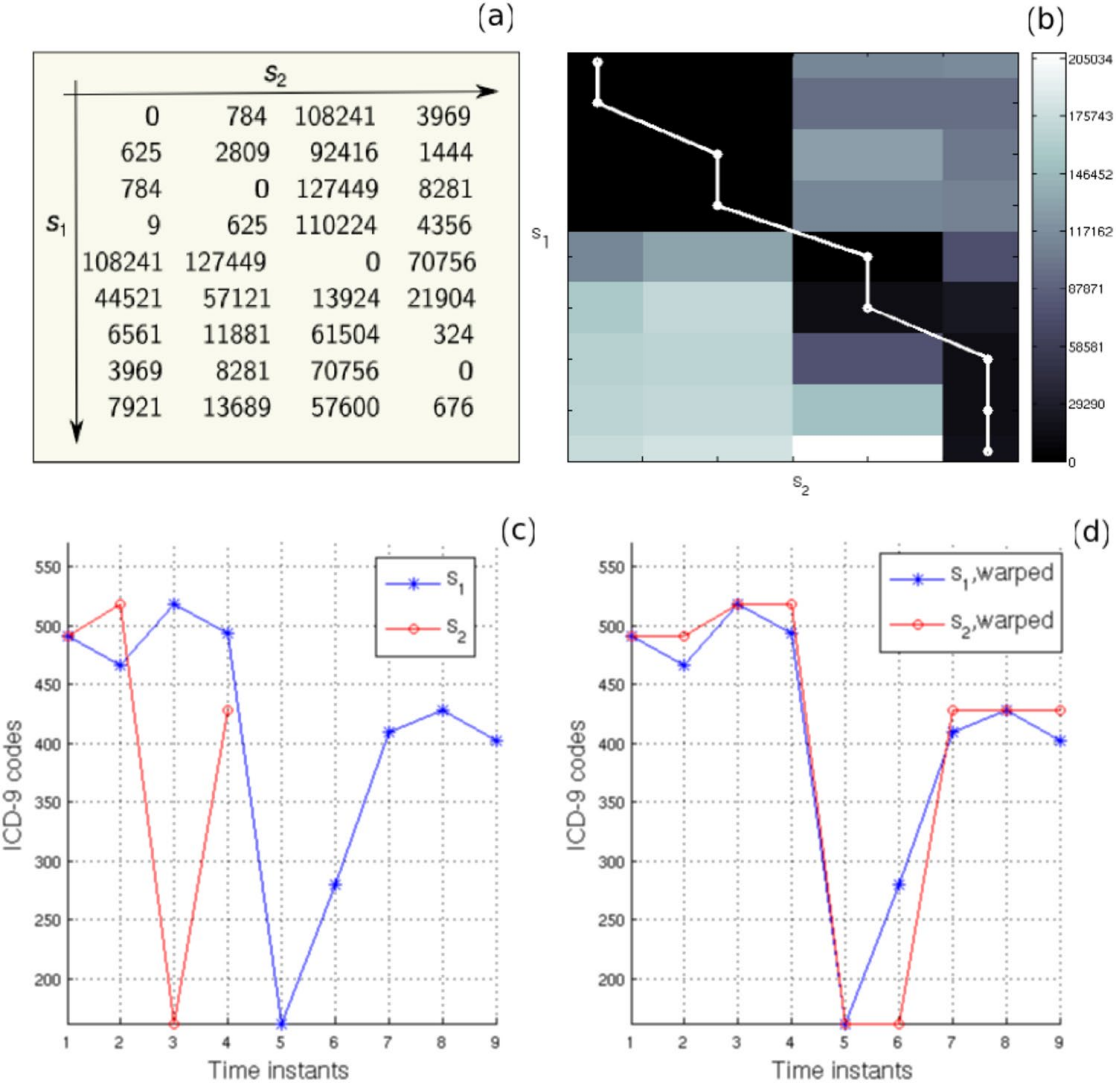
Application of DTW on two disease trajectories. (**a**) A numerical example of the local distance matrix *D* and (**b**) an image of the accumulated distance matrix *A* for two disease trajectories: *s*_1_ = {491, 466, 519, 494, 162, 280, 410, 428, 402} and *s*_2_ = {491, 519, 162, 428}. The optimal path is superimposed in (b) in white. (**c**) The original disease trajectories *s*_1_ and *s*_2_ and (**d**) warped ones after applying the DTW algorithm.

**Figure 5:**
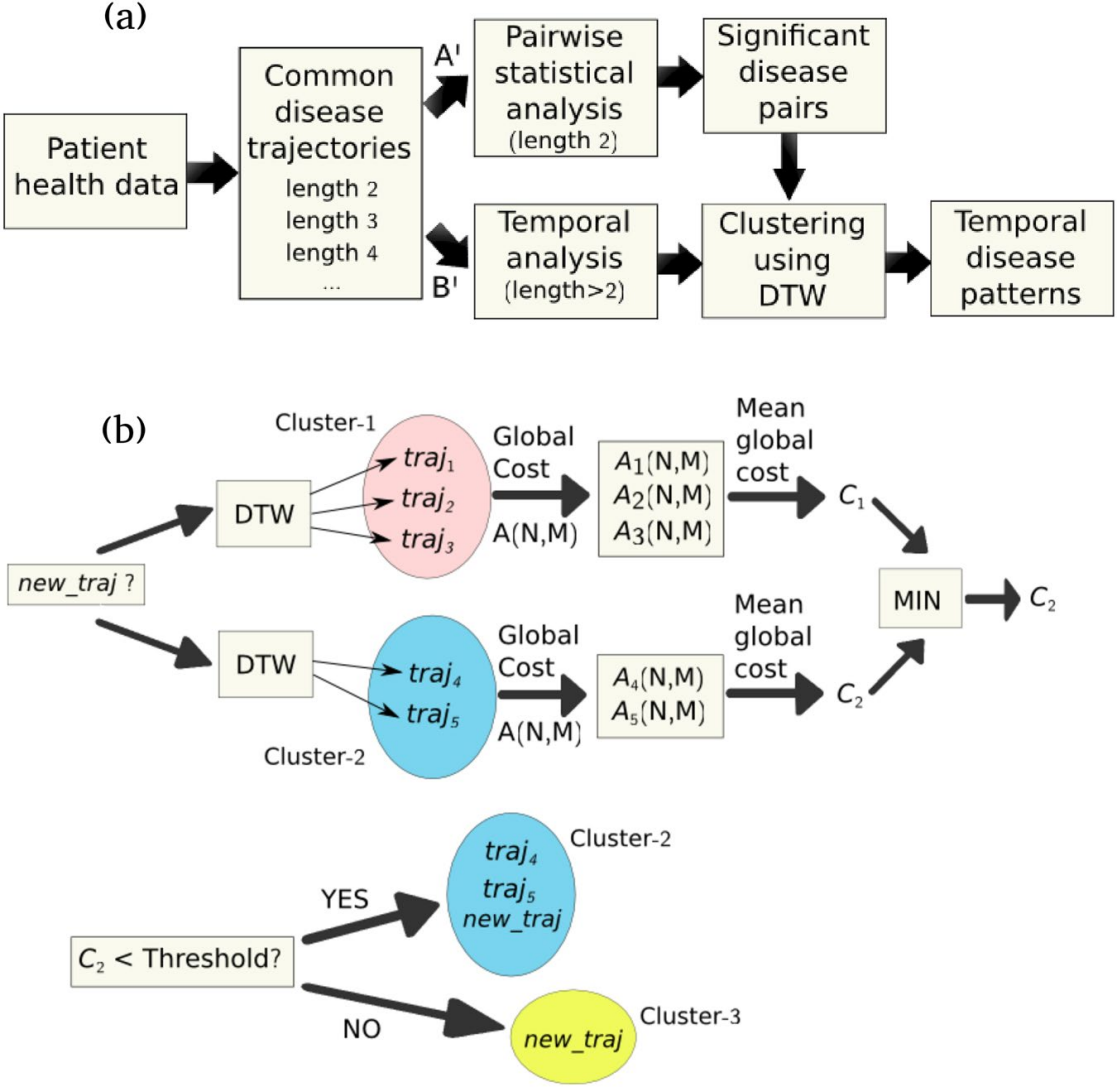
Flow-charts of the proposed methodology. (**a**) A flow-chart of the proposed methodology for the extraction of time-dependent disease associations and (**b**) the unsupervised clustering method of the common disease trajectories using the DTW algorithm. *A*_*i*_(*N*,*M*) denotes the final accumulated distance or global cost between the new incoming trajectory (*new_traj*) and each trajectory *traj*_*i*_ (*i* = i, 2, …) of the existing clusters, according to equation (2).

The above matrix can be regarded as a measure of the local *dissimilarity* (distance) between two time sequences. It will be, initially, used for the identification of identical diseases (“matches”) between the history vectors of different patients in order to extract all common disease trajectories and also, as a distance metric for clustering the extracted trajectories (described in a subsequent section). An example of the local distance matrix *D* for two arbitrary disease-history vectors *s*_1_ = {491, 466, 519, 494, 162, 280, 410, 428, 402} and *s*_2_ = {491, 519, 162, 428} can be seen in Fig. 4a. If two or more zeros are found in *D* (i.e. at least two disease codes of the two history vectors are identical), then those diseases are considered to form a “match”, which will be referred to, in this paper, as *common disease trajectory*. In this manner, lists of common disease trajectories of shared codes of length 2, 3, 4, etc., are obtained separately for men and women. In the example illustrated in Fig. 4a, four zeros are found in *D*, such that, a common disease trajectory of length 4 is extracted, i.e. {491, 519, 162, 428}. A schematic illustration of the workflow of the proposed methodology is presented in Fig. 5a.

### Pairwise statistical comorbidity analysis

In the first part of our proposed analysis, only pairwise comorbidities are examined ignoring all common disease trajectories of length >2 (see Fig. 5a, branch A’). Specifically, disease code pairs (*d*_1_, *d*_2_), in which at least 10 patients share both codes *d*_1_ and *d*_2_, are considered. In order to identify significant disease associations, the Fisher’s exact test^46^ is applied on 2 × 2 contingency tables for each pair and the resulting *p*-values are corrected using the Bonferroni correction for multiple testing.

The temporal direction (*d*_1_ → *d*_2_ and *d*_2_ → *d*_1_) of the pairwise associations, identified as statistically significant in the previous step, is assessed using the binomial test. Specifically, the number of patients for whom, diagnosis *d*_2_ follows diagnosis *d*_1_, or vice versa, is computed and the probability of such figures is calculated using the binomial distribution with a probability of success equal to 0.5. A statistically significant *preferred* direction (the one that appears more often) is assigned for those pairs with *p*-values < 0.05.

### Clustering of common disease trajectories using Dynamic Time Warping

In this part of the study, the extracted common disease trajectories of all lengths (≥2) were considered (see Fig. 5a, branch B’). In order to identify shared temporal patterns, a novel unsupervised clustering algorithm, based on *dynamic time warping* (DTW), is proposed, which aims at grouping the trajectories with similar diagnoses patterns into clusters (according to the ICD-9 hierarchical assignment of diseases), irrespective of their time scale and length. In time series analysis, DTW is a powerful dynamic-programming algorithm for measuring similarities between two temporal sequences that may vary in time or speed^15^. DTW has been successfully used in automatic speech recognition^14^, where the method deals with different speaking speeds or sampling frequencies, as well as, in gene-expression time analysis^47^, hand-writing recognition^48^, gesture recognition^49^, surveillance^50^, financial data analysis^51^, etc. Herewith, the first implementation of DTW on patient disease trajectories is demonstrated.

DTW works by calculating an optimal path (*warping path*) between two given sequences (e.g. times series), with certain restrictions^15^, that minimizes the total distance between them. This distance is calculated according to the *accumulated distance matrix A* (also known as *global cost matrix*), which, in turn, is constructed on the basis of the local distance matrix *D* described in equation (1). Specifically, each element of the *N* × *M* accumulated distance matrix *A* is obtained by adding to the corresponding element of *D*, the minimum of the three previously determined elements of *A*, i.e.:

2 $$A(n,m)=A(n,m)+min\{A(n-1,m),A(n,m-1),A(n-1,m-1)\}$$

The final *accumulated distance* or *global cost* between two sequences can be, thus, determined and is defined by the matrix element *A*(*N*, *M*), i.e., the element of the last row and column of A.

The sequences can be, next, aligned (*warped*) in a non-linear manner along the time dimension, according to the calculated warping path. As mentioned above, the best possible alignment (warping path) is the one that minimizes the final accumulated distance (*A*(*N*, *M*)). This distance will be used in the clustering algorithm described below. Identical signals will result in a diagonal optimal path and a global cost of zero (i.e., *A*(*N*, *M*) = 0), while larger differences between two signals will increase the accumulated distance. An example of the accumulated distance matrix together with the optimal path is shown in Fig. 4b. The original and aligned sequences, according to the extracted warping path, are shown in Fig. 4c and d, respectively. Low values (close to 0) in the local and accumulated distance matrices indicate diseases that, possibly, belong to the same disease group or sub-group (in reference to the ICD-9 hierarchical coding system^45^), while larger values are obtained for more dissimilar diseases (i.e. belonging to different groups or sub-groups as defined by the ICD-9 hierarchy). The acceptable level of “dissimilarity” between two given disease sequences is defined in the clustering algorithm below with the selection of an appropriate threshold (see below). More sophisticated distance metrics could be, alternatively, used in the DTW algorithm (as commented in Discussion and Conclusions).

Due to the above characteristics, the application of the DTW method for analysing the similarity between the disease trajectories appears as a natural and promising choice, given that the trajectories may span different time intervals but may in fact hide similar disease patterns at different time scales. Moreover, DTW allows the alignment of the disease trajectories under study despite their different lengths (durations) (see Fig. 4c,d). This facilitates the identification of important disease patterns shared by disease trajectories that may contain a different number of diagnoses and have no apparent temporal alignment. Furthermore, the shared diseases may not, necessarily, represent consecutive diagnoses within the investigated trajectories, yet they always appear as an ordered sequence.

The proposed clustering algorithm, illustrated in Fig. 5b, belongs to the class of unsupervised machine learning methods and is described as follows. The first common disease trajectory under investigation is automatically assigned to Cluster 1. Given a new incoming trajectory *new_traj* that needs to be clustered, the mean distance is calculated between *new_traj* and all the members of each existing cluster (e.g. trajectories *traj*_*i*_, *i* = 1, 2, 3 for Cluster 1 and *i* = 4, 5 for Cluster 2). For this purpose, the DTW algorithm is applied between the new and each trajectory of an existing cluster and, as previously discussed, the global cost *A*_*i*_(*N*, *M*), resulting from the accumulated distance matrix *A* according to equation (2), is used as a measure of distance between each pair of trajectories. Subsequently, the mean distance (global cost) between the new trajectory and all members of an existing cluster is calculated and the minimum mean distance is identified. If the minimum mean distance is lower than a predefined threshold, the new trajectory is assigned to the cluster that produces such a minimum average distance. Otherwise, a new cluster is generated that only consists of the new disease trajectory *new_traj*. The threshold has to be appropriately selected, as a compromise between merging of important clusters (for larger values of the threshold) and excessive fragmentation of them (for lower values of the threshold). The clustering process is repeated in the same manner until there are no other trajectories to be assigned to any cluster. A semi-automatic post-refinement step follows in order to check and correct for possible errors that may have resulted from diseases that produce low distance metrics (as defined by the ICD-9 hierarchical coding system), but belong to different neighbouring disease sub-groups^16^. In such cases, the clusters are further split into smaller sub-clusters. Finally, the obtained clusters are manually reviewed in order to decide whether further merging or fragmentation is needed.

## Electronic supplementary material

Supplementary Information
