# Supplementary material for: Identifying temporal patterns in patient disease trajectories using dynamic time warping: A population-based study
Source: Sci Rep. 2018 Mar 9;8:4216. doi: 10.1038/s41598-018-22578-1 (PMC5844976; doi:10.1038/s41598-018-22578-1)
Supplement: Supplementary file 1 — Supplementary Information [file 41598_2018_22578_MOESM1_ESM.pdf]

Identifying temporal patterns in patient disease trajectories using dynamic time warping: A population-based study

Alexia Giannoula, Alba Gutierrez-Sacristán, Álex Bravo, Ferran Sanz and Laura I. Furlong*

Supplementary Table 1. Frequency of certain diseases or groups of diseases (ICD-9 coding) in the male and female sub-populations of the study.

Disease/Disease group (ICD-9 code)	MEN	WOMEN
Neoplasms (140-239)	29.2%	20.8%
-Bladder cancer (188)	5.2%	0.7%
-Colon cancer (153)	2.8%	1.7%
-Breast cancer (174)	-	4.0%
Cataract (366)	22%	23.1%
Diabetes Mellitus (250)	3.2%	2.6%
Diseases of the circulatory system (390-459)	37.9%	30.4%
-Hypertensive disease (401-405)	1.7%	2.1%
-Ischemic heart disease (410-414)	12.3%	5.4%
-Other forms of heart disease (420-429)	14.9%	13.7%
-Atherosclerosis (440)	2.9%	1.0%
-Cerebrovascular disease (430-438)	8.7%	6.3%
Diseases of the respiratory system (460-519)	31.8%	20.9%
-COPD (490-496)	11.2%	5.1%
-Asthma (493)	0.9%	2.7%
Diseases muskulosk sys & conn tissue (710-739)	14.7%	22.0%
-Osteoarthrosis (715)	4.8%	8.4%
-Other disorders of bone and cartilage (733)	1.3%	1.5%
Mental disorders (290-319)	3.5%	2.7%

Supplementary Table 2. Number of disease trajectories considered in the clustering algorithm for various trajectory lengths for the male and female sub-populations of the study. In each case, a minimum number of patients was considered. A total number of 10,245 and 7,553 trajectories were, finally, used for men and women, correspondingly.

Length of trajectory	Patient threshold	#trajectories	
		MEN	WOMEN
2	≥50	1783	1914
3	≥20	4386	3032
4	≥5	3789	2421
5	≥3	244	143
6	≥2	43	43

Supplementary Table 3. Distribution of the number of clusters obtained for men and women as a function of the cluster size (#trajectories within cluster). The average number of patients in each case is also listed. The total number of clusters obtained using the DTW algorithm was 734 and 703, for men and women, correspondingly.

**#trajectories within cluster**	**MEN**		**WOMEN**	
	**#clusters**	**Average #pat**	**#clusters**	**Average #pat**
≥10	199	57.1	164	69.6
≥6 & <10	97	39.0	84	48.3
5	24	34.7	52	40.7
4	59	34.6	49	38.8
3	54	36.8	51	44.2
2	94	25.2	103	23.4
1	207	20.3	200	16.0

Supplementary Table 4. Twenty most populated clusters extracted for the male sub-population, with low-level description (ICD-9 coding) of the disease sub-groups within the trajectories of each cluster. The clusters are ordered according to the number of patients (second column) found in each case. The number of trajectories contained (first column) is also reported.

**#traj**	**#pat**	**Low-level (ICD-9) sub-group disease distribution**
304	48874	Chronic Obstructive Pulmonary Disease And Allied Conditions (43.4%) Other Diseases Of Respiratory System (1.3%) Pneumonia And Influenza (42.8%) Acute Respiratory Infections (10.7%) Pneumoconioses And Other Lung Diseases Due To External Agents (0.3%) Other Diseases Of Upper Respiratory Tract (1.2%) Diseases Of Veins And Lymphatics, And Other Diseases Of Circulatory System (0.3%)
162	40196	Ischemic Heart Disease (16.2%) Other Forms Of Heart Disease (47.3%) Diseases Of Arteries, Arterioles, And Capillaries (12.9%) Hypertensive Disease (1.5%) Cerebrovascular Disease (17.2%) Chronic Rheumatic Heart Disease (1.5%) Diseases Of Veins And Lymphatics, And Other Diseases Of Circulatory System (2.3%) Diseases Of Pulmonary Circulation (1.0%)
132	22437	Other Diseases Of Urinary System (37.4%) Diseases Of Male Genital Organs (12.6%) Nephritis, Nephrotic Syndrome, And Nephrosis (13.2%) Other Diseases Of Digestive System (32.9%) Other Diseases Of Intestines And Peritoneum (3.9%)
238	18648	Chronic Obstructive Pulmonary Disease And Allied Conditions (26.0%) Other Forms Of Heart Disease (27.9%) Pneumonia And Influenza (19.7%) Acute Respiratory Infections (3.0%) Other Diseases Of Respiratory System (2.3%) Diseases Of Pulmonary Circulation (1.0%) Other Diseases Of Upper Respiratory

		Tract (0.5%) Hypertensive Disease (2.1%) Ischemic Heart Disease (14.1%) Cerebrovascular Disease (2.1%) Diseases Of Arteries, Arterioles, And Capillaries (1.3%)
155	17732	Other Forms Of Heart Disease (21.1%) Other Diseases Of Respiratory System (25.4%) Cerebrovascular Disease (4.7%) Pneumoconioses And Other Lung Diseases Due To External Agents (3.6%) Diseases Of Veins And Lymphatics, And Other Diseases Of Circulatory System (0.4%) Chronic Obstructive Pulmonary Disease And Allied Conditions (6.8%) Ischemic Heart Disease (32.3%) Pneumonia And Influenza (3.6%) Hypertensive Disease (1.3%) Diseases Of Pulmonary Circulation (0.4%) Diseases Of Arteries, Arterioles, And Capillaries (0.4%)
221	16961	Disorders Of The Eye And Adnexa (32.7%) Other Forms Of Heart Disease (23.8%) Disorders Of The Peripheral Nervous System (1.1%) Other Disorders Of The Central Nervous System (0.1%) Diseases Of The Ear And Mastoid Process (0.1%) Cerebrovascular Disease (6.6%) Ischemic Heart Disease (28.8%) Diseases Of Arteries, Arterioles, And Capillaries (3.8%) Hypertensive Disease (2.7%) Chronic Rheumatic Heart Disease (0.1%)
192	13557	Hypertensive Disease (2.1%) Nephritis, Nephrotic Syndrome, And Nephrosis (12.4%) Other Forms Of Heart Disease (18.6%) Ischemic Heart Disease (32.4%) Other Diseases Of Digestive System (12.7%) Other Diseases Of Urinary System (11.7%) Cerebrovascular Disease (3.7%) Other Diseases Of Intestines And Peritoneum (1.4%) Diseases Of Arteries, Arterioles, And Capillaries (1.6%) Hernia Of Abdominal Cavity (0.2%) Noninfective Enteritis And Colitis (0.2%) Diseases Of Male Genital Organs (3.0%)
142	12700	Disorders Of The Eye And Adnexa (30.7%) Acute Respiratory Infections (7.2%) Disorders Of The Peripheral Nervous System (0.6%) Chronic Obstructive Pulmonary Disease And Allied Conditions (30.0%) Other Diseases Of Upper Respiratory Tract (0.2%) Pneumonia And Influenza (29.0%) Diseases Of Veins And Lymphatics, And Other Diseases Of Circulatory System (0.4%) Other Disorders Of The Central Nervous System (0.2%) Diseases Of The Ear And Mastoid Process (0.2%) Other Diseases Of Respiratory System (0.4%) Pneumoconioses And Other Lung Diseases Due To External Agents (1.1%)
45	11379	Disorders Of The Eye And Adnexa (42.5%) Other Diseases Of Digestive System (30.2%) Appendicitis (0.9%) Other Diseases Of Intestines And Peritoneum (4.7%) Other Diseases Of Urinary System (11.3%) Diseases Of The Ear And Mastoid Process (0.9%) Diseases Of Male Genital Organs (1.9%) Disorders Of The Peripheral Nervous System (0.9%) Noninfective Enteritis And Colitis (2.8%) Nephritis, Nephrotic Syndrome, And Nephrosis (2.8%) Other Disorders Of The Central Nervous System (0.9%)
61	10191	Hernia Of Abdominal Cavity (19.4%) Chronic Obstructive Pulmonary Disease And Allied Conditions (18.7%) Other Diseases Of Respiratory System (15.8%) Other Diseases Of Intestines And Peritoneum (4.3%) Pneumonia And Influenza (15.8%) Other Diseases Of Digestive System (17.3%) Pneumoconioses And Other Lung Diseases Due To External Agents (2.2%) Appendicitis (2.2%) Noninfective Enteritis And Colitis (2.9%) Other Diseases Of Upper Respiratory Tract (1.4%)
233	10096	Other Diseases Of Upper Respiratory Tract (0.5%) Diseases Of Oral Cavity, Salivary Glands, And Jaws (0.1%) Acute Respiratory Infections (10.0%) Other

		Diseases Of Respiratory System (29.0%) Chronic Obstructive Pulmonary Disease And Allied Conditions (28.6%) Appendicitis (0.5%) Diseases Of Veins And Lymphatics, And Other Diseases Of Circulatory System (0.1%) Pneumonia And Influenza (25.4%) Pneumoconioses And Other Lung Diseases Due To External Agents (4.9%) Diseases Of Esophagus, Stomach, And Duodenum (0.4%) Hernia Of Abdominal Cavity (0.4%)
223	9732	Ischemic Heart Disease (52.6%) Acute Respiratory Infections (0.7%) Diseases Of Arteries, Arterioles, And Capillaries (6.2%) Cerebrovascular Disease (5.2%) Chronic Rheumatic Heart Disease (0.1%) Other Forms Of Heart Disease (32.5%) Hypertensive Disease (2.0%) Diseases Of Veins And Lymphatics, And Other Diseases Of Circulatory System (0.7%)
51	9405	Disorders Of The Eye And Adnexa (94.4%) Diseases Of The Ear And Mastoid Process (3.2%) Other Disorders Of The Central Nervous System (1.6%) Pain (0.8%)
37	8298	Hereditary And Degenerative Diseases Of The Central Nervous System (2.1%) Pneumoconioses And Other Lung Diseases Due To External Agents (6.2%) Disorders Of The Eye And Adnexa (37.5%) Other Diseases Of Respiratory System (34.4%) Disorders Of The Peripheral Nervous System (1.0%) Other Disorders Of The Central Nervous System (1.0%) Diseases Of Esophagus, Stomach, And Duodenum (2.1%) Diseases Of Oral Cavity, Salivary Glands, And Jaws (1.0%) Pneumonia And Influenza (5.2%) Chronic Obstructive Pulmonary Disease And Allied Conditions (9.4%)
63	8243	Malignant Neoplasm Of Genitourinary Organs (34.9%) Other Diseases Of Urinary System (40.7%) Benign Neoplasms (1.2%) Other Diseases Of Intestines And Peritoneum (1.7%) Other Diseases Of Digestive System (4.7%) Nephritis, Nephrotic Syndrome, And Nephrosis (11.0%) Malignant Neoplasm Of Other And Unspecified Sites (1.7%) Noninfective Enteritis And Colitis (1.2%) Diseases Of Male Genital Organs (2.3%) Malignant Neoplasm Of Bone, Connective Tissue, Skin, And Breast (0.6%)
57	7871	Hernia Of Abdominal Cavity (25.0%) Diseases Of Male Genital Organs (2.4%) Other Diseases Of Intestines And Peritoneum (20.2%) Other Diseases Of Digestive System (29.0%) Diseases Of Esophagus, Stomach, And Duodenum (7.3%) Noninfective Enteritis And Colitis (8.1%) Other Diseases Of Respiratory System (0.8%) Nephritis, Nephrotic Syndrome, And Nephrosis (3.2%) Other Diseases Of Urinary System (3.2%) Appendicitis (0.8%)
120	7292	Cerebrovascular Disease (5.8%) Other Disorders Of The Central Nervous System (1.3%) Diseases Of Veins And Lymphatics, And Other Diseases Of Circulatory System (0.3%) Disorders Of The Eye And Adnexa (30.4%) Other Forms Of Heart Disease (18.6%) Ischemic Heart Disease (39.7%) Diseases Of Arteries, Arterioles, And Capillaries (3.5%) Hypertensive Disease (0.5%)
64	7249	Other Diseases Of Digestive System (38.8%) Diseases Of Esophagus, Stomach, And Duodenum (6.8%) Diseases Of Male Genital Organs (2.0%) Hernia Of Abdominal Cavity (15.6%) Other Diseases Of Intestines And Peritoneum (21.1%) Nephritis, Nephrotic Syndrome, And Nephrosis (2.7%) Noninfective Enteritis And Colitis (9.5%) Other Diseases Of Urinary System (2.7%) Appendicitis (0.7%)
23	6936	Disorders Of The Eye And Adnexa (41.5%) Malignant Neoplasm Of Other And

		Unspecified Sites (20.8%) Malignant Neoplasm Of Respiratory And Intrathoracic Organs (9.4%) Malignant Neoplasm Of Bone, Connective Tissue, Skin, And Breast (1.9%) Malignant Neoplasm Of Lymphatic And Hematopoietic Tissue (9.4%) Disorders Of The Peripheral Nervous System (1.9%) Malignant Neoplasm Of Genitourinary Organs (13.2%) Benign Neoplasms (1.9%)
56	6748	Diseases Of Male Genital Organs (17.3%) Other Diseases Of Upper Respiratory Tract (1.4%) Other Diseases Of Urinary System (23.0%) Pneumoconioses And Other Lung Diseases Due To External Agents (5.0%) Chronic Obstructive Pulmonary Disease And Allied Conditions (15.8%) Other Diseases Of Respiratory System (12.2%) Acute Respiratory Infections (5.0%) Pneumonia And Influenza (15.8%) Nephritis, Nephrotic Syndrome, And Nephrosis (3.6%) Other Diseases Of Digestive System (0.7%)

Supplementary Table 5. Twenty most populated clusters extracted for the female sub-population, with low-level description (ICD-9 coding) of the disease sub-groups within the trajectories of a cluster. The clusters are ordered according to the number of patients (second column) found in each case. The number of trajectories contained (first column) is also reported.

#traj	#pat	Low-level (icd9) sub-group disease distribution
374	58672	Normal Delivery, And Other Indications For Care In Pregnancy, Labor, And Delivery (29.6%) Complications Occurring Mainly In The Course Of Labor And Delivery (17.6%) Complications Mainly Related To Pregnancy (32.4%) Ectopic And Molar Pregnancy And Other Pregnancy With Abortive Outcome (18.0%) Complications Of The Puerperium (1.1%) Other Disorders Of Female Genital Tract (1.4%)
220	29781	Acute Respiratory Infections (12.2%) Chronic Obstructive Pulmonary Disease And Allied Conditions (33.5%) Other Diseases Of Respiratory System (22.9%) Pneumonia And Influenza (26.9%) Pneumoconioses And Other Lung Diseases Due To External Agents (4.1%) Other Diseases Of Upper Respiratory Tract (0.3%)
301	28588	Ischemic Heart Disease (53.5%) Other Forms Of Heart Disease (32.0%) Chronic Rheumatic Heart Disease (1.8%) Hypertensive Disease (7.8%) Diseases Of Arteries, Arterioles, And Capillaries (0.3%) Diseases Of Pulmonary Circulation (0.5%) Cerebrovascular Disease (3.9%) Diseases Of The Ear And Mastoid Process (0.1%)
160	20430	Disorders Of The Peripheral Nervous System (2.9%) Other Forms Of Heart Disease (28.5%) Acute Respiratory Infections (0.8%) Cerebrovascular Disease (9.9%) Disorders Of The Eye And Adnexa (32.8%) Diseases Of Veins And Lymphatics, And Other Diseases Of Circulatory System (0.6%) Ischemic Heart Disease (17.3%) Diseases Of Arteries, Arterioles, And Capillaries (2.5%) Diseases Of The Ear And Mastoid Process (0.4%) Other Disorders Of The Central Nervous System (0.2%) Diseases Of Pulmonary Circulation (0.6%) Hypertensive Disease (3.3%) Chronic Rheumatic Heart Disease (0.2%)
93	17273	Other Forms Of Heart Disease (35.8%) Pneumonia And Influenza (4.7%) Other Diseases Of Respiratory System (26.1%) Chronic Obstructive Pulmonary Disease And Allied Conditions (5.1%) Hypertensive Disease (2.7%) Diseases Of Veins And Lymphatics, And Other Diseases Of Circulatory System (0.4%) Cerebrovascular Disease (6.2%) Pneumoconioses And Other Lung Diseases Due To External Agents

		(6.2%) Diseases Of Pulmonary Circulation (2.3%) Ischemic Heart Disease (9.3%) Diseases Of Esophagus, Stomach, And Duodenum (1.2%)
97	16473	Other Forms Of Heart Disease (50.4%) Acute Respiratory Infections (3.0%) Cerebrovascular Disease (26.3%) Diseases Of Veins And Lymphatics, And Other Diseases Of Circulatory System (4.7%) Diseases Of Arteries, Arterioles, And Capillaries (9.1%) Ischemic Heart Disease (5.2%) Diseases Of Pulmonary Circulation (1.3%)
51	14427	Arthropathies And Related Disorders (26.1%) Disorders Of The Eye And Adnexa (37.7%) Disorders Of The Peripheral Nervous System (9.4%) Osteopathies, Chondropathies, And Acquired Musculoskeletal Deformities (12.3%) Dorsopathies (3.6%) Other Disorders Of The Central Nervous System (0.7%) Rheumatism, Excluding The Back (9.4%) Other Diseases Of Skin And Subcutaneous Tissue (0.7%)
75	13234	Osteopathies, Chondropathies, And Acquired Musculoskeletal Deformities (20.1%) Rheumatism, Excluding The Back (23.6%) Arthropathies And Related Disorders (40.8%) Dorsopathies (15.5%)
68	12598	Other Diseases Of Intestines And Peritoneum (28.8%) Other Diseases Of Digestive System (41.8%) Hernia Of Abdominal Cavity (10.3%) Diseases Of Esophagus, Stomach, And Duodenum (1.4%) Noninfective Enteritis And Colitis (13.0%) Other Diseases Of Urinary System (1.4%) Nephritis, Nephrotic Syndrome, And Nephrosis (2.7%) Appendicitis (0.7%)
130	12373	Pneumonia And Influenza (15.3%) Other Forms Of Heart Disease (29.8%) Chronic Obstructive Pulmonary Disease And Allied Conditions (10.2%) Acute Respiratory Infections (16.6%) Hypertensive Disease (13.8%) Other Diseases Of Respiratory System (1.3%) Diseases Of Pulmonary Circulation (2.0%) Chronic Rheumatic Heart Disease (1.3%) Ischemic Heart Disease (8.7%) Cerebrovascular Disease (1.0%)
74	12056	Other Forms Of Heart Disease (28.4%) Nephritis, Nephrotic Syndrome, And Nephrosis (9.3%) Other Diseases Of Urinary System (8.2%) Cerebrovascular Disease (2.7%) Other Diseases Of Digestive System (15.8%) Hypertensive Disease (7.1%) Noninfective Enteritis And Colitis (8.2%) Other Diseases Of Intestines And Peritoneum (2.2%) Ischemic Heart Disease (15.8%) Diseases Of Arteries, Arterioles, And Capillaries (1.1%) Diseases Of Pulmonary Circulation (0.5%) Hernia Of Abdominal Cavity (0.5%)
42	11133	Disorders Of The Eye And Adnexa (90.2%) Other Disorders Of The Central Nervous System (2.2%) Diseases Of The Ear And Mastoid Process (3.3%) Disorders Of The Peripheral Nervous System (2.2%) Hypertensive Disease (1.1%) Pain (1.1%)
62	7843	Osteopathies, Chondropathies, And Acquired Musculoskeletal Deformities (9.3%) Other Forms Of Heart Disease (25.2%) Acute Respiratory Infections (2.6%) Arthropathies And Related Disorders (25.2%) Rheumatism, Excluding The Back (4.6%) Other Diseases Of Skin And Subcutaneous Tissue (0.7%) Diseases Of Arteries, Arterioles, And Capillaries (1.3%) Cerebrovascular Disease (6.6%) Ischemic Heart Disease (18.5%) Dorsopathies (2.0%) Diseases Of Pulmonary Circulation (0.7%) Hypertensive Disease (2.0%) Diseases Of Veins And Lymphatics, And Other Diseases Of Circulatory System (1.3%)
41	7652	Disorders Of The Eye And Adnexa (33.3%) Other Diseases Of Digestive System (28.7%) Disorders Of The Peripheral Nervous System (7.4%) Noninfective Enteritis And Colitis (4.6%) Other Diseases Of Intestines And Peritoneum (12.0%) Nephritis, Nephrotic Syndrome, And Nephrosis (3.7%) Other Diseases Of Urinary System (6.5%) Hernia Of Abdominal Cavity (3.7%)
45	7010	Other Diseases Of Urinary System (65.7%) Nephritis, Nephrotic Syndrome, And Nephrosis (14.7%) Noninfective Enteritis And Colitis (1.0%) Other Diseases Of Digestive System (13.7%) Other Diseases Of Intestines And Peritoneum (2.9%) Other Disorders Of Female Genital Tract (2.0%)

135	6464	Ischemic Heart Disease (20.0%) Acute Respiratory Infections (20.0%) Cerebrovascular Disease (2.5%) Diseases Of Pulmonary Circulation (0.2%) Other Forms Of Heart Disease (28.4%) Diseases Of Veins And Lymphatics, And Other Diseases Of Circulatory System (0.5%) Diseases Of Arteries, Arterioles, And Capillaries (0.7%) Hypertensive Disease (5.7%) Pneumonia And Influenza (15.1%) Chronic Obstructive Pulmonary Disease And Allied Conditions (6.9%)
24	5796	Acute Respiratory Infections (17.6%) Other Diseases Of Urinary System (7.8%) Diseases Of Veins And Lymphatics, And Other Diseases Of Circulatory System (25.5%) Other Diseases Of Intestines And Peritoneum (11.8%) Pneumonia And Influenza (3.9%) Other Diseases Of Digestive System (17.6%) Noninfective Enteritis And Colitis (5.9%) Hernia Of Abdominal Cavity (7.8%) Nephritis, Nephrotic Syndrome, And Nephrosis (2.0%)
49	5754	Disorders Of The Peripheral Nervous System (19.5%) Rheumatism, Excluding The Back (15.0%) Arthropathies And Related Disorders (23.3%) Osteopathies, Chondropathies, And Acquired Musculoskeletal Deformities (12.0%) Disorders Of The Eye And Adnexa (24.8%) Dorsopathies (4.5%) Other Diseases Of Skin And Subcutaneous Tissue (0.8%)
22	5359	Disorders Of The Eye And Adnexa (29.4%) Other Disorders Of Female Genital Tract (7.8%) Disorders Of The Peripheral Nervous System (13.7%) Other Diseases Of Urinary System (37.3%) Disorders Of Breast (2.0%) Ectopic And Molar Pregnancy And Other Pregnancy With Abortive Outcome (2.0%) Hereditary And Degenerative Diseases Of The Central Nervous System (2.0%) Other Disorders Of The Central Nervous System (2.0%) Nephritis, Nephrotic Syndrome, And Nephrosis (3.9%)
63	5332	Disorders Of The Peripheral Nervous System (2.9%) Pneumonia And Influenza (27.5%) Disorders Of The Eye And Adnexa (28.4%) Pneumoconioses And Other Lung Diseases Due To External Agents (1.5%) Chronic Obstructive Pulmonary Disease And Allied Conditions (24.0%) Other Disorders Of The Central Nervous System (0.5%) Hereditary And Degenerative Diseases Of The Central Nervous System (0.5%) Acute Respiratory Infections (13.7%) Other Diseases Of Respiratory System (1.0%)

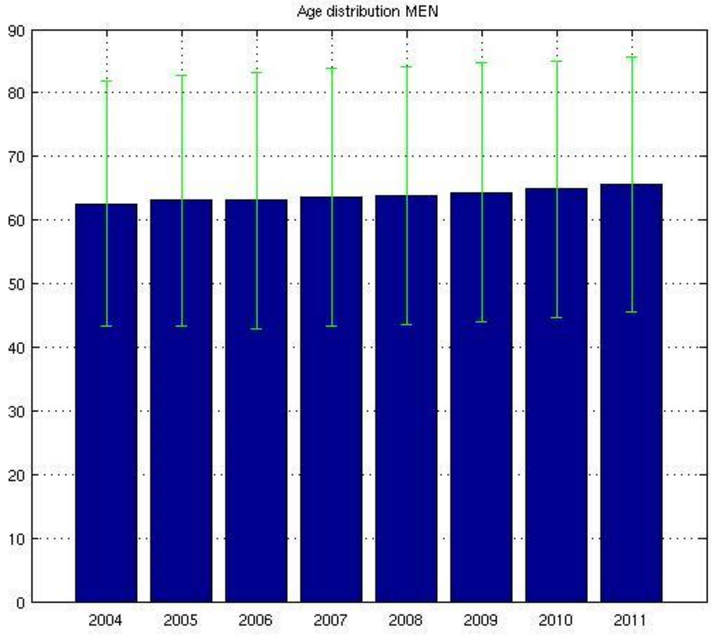

(a)

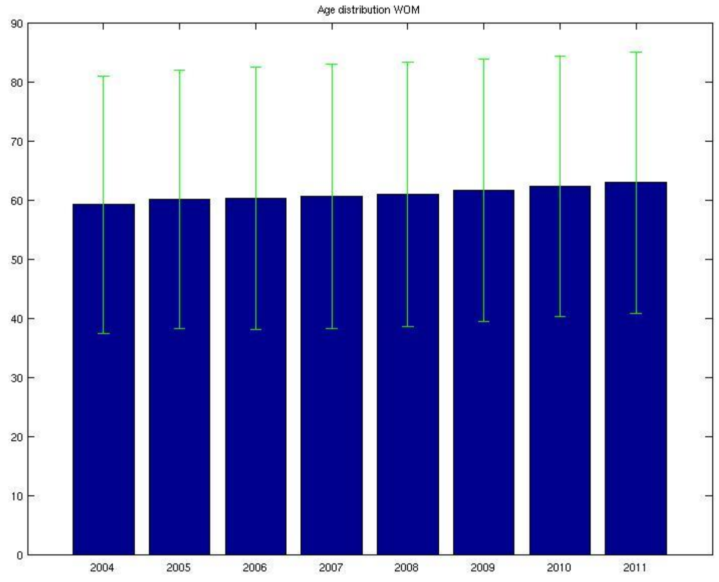

(b)

Supplementary Figure 1. **Age distribution of the male and female patients.** The mean age of patients (together with the corresponding standard deviation) for each year of hospitalization, for the (a) male and (b) female sub-populations.
